# Randomized phase II trial of MRI-guided salvage radiotherapy for prostate cancer in 4 weeks versus 2 weeks (SHORTER)

**DOI:** 10.1186/s12885-023-11278-3

**Published:** 2023-08-22

**Authors:** Ariel E. Marciscano, Sydney Wolfe, Xi Kathy Zhou, Christopher E. Barbieri, Silvia C. Formenti, Jim C. Hu, Ana M. Molina, David M. Nanus, Jones T. Nauseef, Douglas S. Scherr, Cora N. Sternberg, Scott T. Tagawa, Himanshu Nagar

**Affiliations:** 1 Department of Radiation Oncology, Weill Cornell Medicine/NewYork-Presbyterian, 525 East 68th Street, Box 169, New York, NY N-046 USA; 2 Department of Population Health Sciences, Division of Biostatistics, Weill Cornell Medicine/NewYork-Presbyterian, New York, NY USA; 3 Department of Urology, Weill Cornell Medicine/NewYork-Presbyterian, New York, NY USA; 4 Sandra and Edward Meyer Cancer Center, Weill Cornell Medicine/NewYork-Presbyterian, New York, NY USA; 5 Englander Institute for Precision Medicine, Weill Cornell Medicine/New York-Presbyterian, New York, NY USA; 6 Division of Hematology/Oncology, Department of Medicine, Weill Cornell Medicine/NewYork-Presbyterian, New York, NY USA

**Keywords:** Prostate cancer, Salvage radiotherapy, Stereotactic body radiotherapy (SBRT), MR-Linac, MRI-guided radiotherapy (MRgRT), Genitourinary, Hypofractionation, Ultra-hypofractionated, Toxicity

## Abstract

**Background:**

Ultra-hypofractionated image-guided stereotactic body radiotherapy (SBRT) is increasingly used for definitive treatment of localized prostate cancer. Magnetic resonance imaging-guided radiotherapy (MRgRT) facilitates improved visualization, real-time tracking of targets and/or organs-at-risk (OAR), and capacity for adaptive planning which may translate to improved targeting and reduced toxicity to surrounding tissues. Given promising results from NRG-GU003 comparing conventional and moderate hypofractionation in the post-operative setting, there is growing interest in exploring ultra-hypofractionated post-operative regimens. It remains unclear whether this can be done safely and whether MRgRT may help mitigate potential toxicity. SHORTER (NCT04422132) is a phase II randomized trial prospectively evaluating whether salvage MRgRT delivered in 5 fractions versus 20 fractions is non-inferior with respect to gastrointestinal (GI) and genitourinary (GU) toxicities at 2-years post-treatment.

**Methods:**

A total of 136 patients will be randomized in a 1:1 ratio to salvage MRgRT in 5 fractions or 20 fractions using permuted block randomization. Patients will be stratified according to baseline Expanded Prostate Cancer Index Composite (EPIC) bowel and urinary domain scores as well as nodal treatment and androgen deprivation therapy (ADT). Patients undergoing 5 fractions will receive a total of 32.5 Gy over 2 weeks and patients undergoing 20 fractions will receive a total of 55 Gy over 4 weeks, with or without nodal coverage (25.5 Gy over 2 weeks and 42 Gy over 4 weeks) and ADT as per the investigator’s discretion. The co-primary endpoints are change scores in the bowel and the urinary domains of the EPIC. The change scores will reflect the 2-year score minus the pre-treatment (baseline) score. The secondary endpoints include safety endpoints, including change in GI and GU symptoms at 3, 6, 12 and 60 months from completion of treatment, and efficacy endpoints, including time to progression, prostate cancer specific survival and overall survival.

**Discussion:**

The SHORTER trial is the first randomized phase II trial comparing toxicity of ultra-hypofractionated and hypofractionated MRgRT in the salvage setting. The primary hypothesis is that salvage MRgRT delivered in 5 fractions will not significantly increase GI and GU toxicities when compared to salvage MRgRT delivered in 20 fractions.

**Trial registration:**

ClinicalTrials.gov Identifier: NCT04422132. Date of registration: June 9, 2020.

**Supplementary Information:**

The online version contains supplementary material available at 10.1186/s12885-023-11278-3.

## Background

Prostate cancer is the most common non-cutaneous cancer and the second leading cause of cancer death in men [1]. It is predicted that the number of prostate cancer cases will almost double by the year 2030 [2]. For men with localized prostate cancer, radical prostatectomy is a common definitive treatment modality [3]. After radical prostatectomy, approximately one-third to one-half of men with high-risk features will develop biochemical recurrence (BCR) [4–7].

Post-operative radiotherapy is the current standard of care for men who develop BCR or are at risk of BCR after radical prostatectomy. Post-operative radiotherapy is a potentially curative treatment after prostatectomy for men with BCR and may avoid or delay the need for chronic, non-curative treatment, such as long-term androgen suppression. Given the increase in both prostate cancer diagnosis and the proportion of men with high-risk disease undergoing radical prostatectomy, the number of men requiring post-operative radiotherapy is likely to increase [8]. As such, optimizing radiotherapy approaches for post-operative management is an important and unanswered question. Minimizing genitourinary (GU) and gastrointestinal (GI) toxicity and maximizing quality of life are critical for men with a high chance of cure and long life-expectancy following salvage treatment [9].

Adjuvant radiotherapy, the administration of immediate post-operative radiotherapy based on adverse pathological features, has been demonstrated to improve biochemical progression free survival (PFS) across multiple prospective randomized trials [10–12]. More recently, early salvage post-operative radiotherapy has become an accepted paradigm. In this approach, men delay post-operative intervention until the time of BCR or if there is a persistently elevated serum prostate-specific antigen (PSA) level after prostatectomy. In support of this paradigm shift, three recent randomized controlled trials and the harmonized ARTISTIC meta-analysis have demonstrated that adjuvant radiotherapy, as compared with salvage radiotherapy, does not improve event-free survival [4, 5, 7, 13]. Additionally, the salvage approach may also avoid overtreatment in the subset of men that despite high-risk features do not develop BCR. The aforementioned studies have helped clarify the timing of post-operative intervention, however, integration of genomic classifiers, optimal target volumes (including pelvic nodal coverage), and the use and duration of androgen suppression therapy remain controversial. Additional studies are needed to further refine patient selection and improve post-operative management.

Increasingly, there has been a trend toward delivering higher doses of radiation over fewer treatment sessions, termed hypofractionation, as opposed to longer courses of treatment with conventional fractionation (1.8–2 Gy/fraction) [14–16]. Further, as prostate cancer is believed to have a low α/β ratio relative to other tumor types, the therapeutic ratio may favor hypofractionation over conventional regimens [17–20]. The preliminary report of the phase III randomized NRG-GU003 study demonstrated that moderately hypofractionated post-operative radiotherapy (62.5 Gy in 25 fractions) does not increase patient-reported GU or GI toxicity over conventional post-operative radiotherapy (66.6 Gy in 37 fractions). At a median of 24 months, change in mean GU and GI scores in the moderate hypofractionation arm and conventional fractionation arm were neither clinically nor statistically significant (mean GU = -5.2 vs mean GU = -0.3, p= 0.81; mean GI = -2.2 vs mean GI = -1.5, *p* = 0.12). Therefore, moderate hypofractionation is non-inferior to conventional fractionation for post-operative RT in measures of late toxicity [21]. This finding is significant, as hypofractionation provides patients with shorter and more accessible courses of radiotherapy, thereby reducing treatment burden. 

The advent of magnetic resonance imaging (MRI)-guided radiotherapy (MRgRT) – enabled through the integration of MRI and linear accelerator (MR-LINAC) technology may facilitate safe delivery of hypofractionated and ultra-hypofractionated regimens. Multi-parametric MR imaging on the MR-LINAC improves target delineation and this feature combined with real-time MR image-guidance and tracking, allows for reduction in planning margins and accounts for inter-fractional changes in anatomy during treatment thereby decreasing dose to adjacent structures, including the bladder and rectum [22]. In addition, the MR-LINAC technology can facilitate online adaptive planning as needed. Taken together, MRgRT may safely allow larger radiation doses to be delivered in fewer fractions in the intact and post-operative setting. The MIRAGE phase III study comparing MRI-guidance versus standard computed tomography (CT)-guidance with stereotactic body radiotherapy (SBRT) for intact prostate cancer demonstrated that acute grade ≥ 2 GU toxicity was significantly reduced among men receiving MRgRT (43.4.1% vs. 24.4%, *p* = 0.01). Acute grade ≥ 2 GI toxicity was also significantly reduced in men receiving MRgRT (10.5% vs. 0%, *p* = 0.003) [23]. Similarly, the SCIMITAR phase II study evaluated the safety profile of post-prostatectomy SBRT and incorporated a pre-planned, non-randomized exploratory analysis of toxicity and patient-reported quality-of-life (QoL) outcomes between MRI-guidance versus CT-guidance. Compared with CT-guided post-operative SBRT, MRgRT was associated with significantly lower rates of any-grade acute GI toxicity (41.9% vs. 72.5%, *p* = 0.0056) per CTCAE version 4.0 criteria, corresponding to an estimated absolute reduction of 30.5%. In terms of late effects, MRgRT was associated with numerically lower any-grade late GI toxicity, however, these differences were not significantly different (37.7% vs. 29%, *p* = 0.4) when compared with CT-guidance. There were no differences in acute or late GU toxicity in the comparison of patients treated with CT versus MRI-guided SBRT. Overall, three patients experienced grade 3 toxicity (GU, *n* = 1; GI *n* = 2), notably no patients treated with MRgRT experienced any grade 3 toxicity or grade ≥ 2 GI toxicity. While additional randomized studies are needed, these findings demonstrate the potential of MRgRT to improve the precision and safety of post-operative radiation delivery [24].

The SHORTER trial (NCT04422132) is designed to evaluate if MR-guided ultra-hypofractionated post-operative radiotherapy (5 fractions in two weeks) has a non-inferior GU and GI toxicity profile as compared with MR-guided moderately-hypofractionated post-operative radiotherapy (20 fractions in four weeks) among prostate cancer patients undergoing salvage radiotherapy. This randomized phase II study lays the foundation to potentially redefine the standard of care for post-prostatectomy patients with BCR to include MRgRT and decrease treatment burden.

## Methods/design

### Objectives

The primary objective is to demonstrate that 5 fractions of ultra-hypofractionated MRgRT does not significantly increase patient-reported GI and GU symptoms as compared with 20 days of hypofractionated MRgRT at 2 years after treatment completion.

#### Primary endpoint

The co-primary endpoints are change scores in the bowel (GI) or urinary (GU) domains of the Expanded Prostate Cancer Index Composite (EPIC). The change scores will reflect the 2-year score minus the pre-treatment (baseline) score.

#### Secondary endpoints

- 1. Compare patient-reported GI symptoms using the EPIC questionnaire at the end of RT and 3, 6, 12, and 60 months from end of treatment.
- 2. Compare patient-reported GU symptoms using the EPIC questionnaire at the end of RT and 3, 6, 12, and 60 months from end of treatment.
- 3. Compare time to progression (TTP) where progression is defined as the first occurrence of biochemical failure (BF), local failure, regional failure, distant metastasis (DM), institution of new unplanned anticancer treatment, or death from prostate cancer (PCSM).
- 4. Compare freedom from biochemical failure (FFBF) and TTP rates with an alternate PSA ≥ PSA nadir + 2 ng/mL definition of BF.
- 5. Compare local failure, regional failure, salvage therapy (*i.e.,* institution of new unplanned anticancer treatment), DM, PCSM, and overall survival (OS) rates.

#### Inclusion criteria

- Men aged ≥ 18 with histologically confirmed prostate cancer after prostatectomy with detectable PSA. Patients with detectable post-prostatectomy PSA whether (1) persistently detectable post-operatively or (2) developing biochemical recurrence after prostatectomy (initially undetectable) are eligible. Patients with early BCR or persistently detectable PSA after prostatectomy must wait a minimum of 6 months post-prostatectomy but can initiate ADT as indicated. PSA does not need to be detectable for men with pathologically node positive disease.
- KPS ≥ 70
- Patient with no evidence of distant metastatic disease on positron emission tomography (PET)/CT/MRI or bone scan < 90–180 days prior to enrollment. Patients with positive pelvic lymph nodes are eligible.
- Ability to receive MRI-guided radiotherapy
- Equivocal evidence of metastatic disease outside the pelvis on standard imaging requires documented negative biopsy
- Ability to complete the EPIC questionnaire

#### Exclusion criteria

- Prior history of receiving pelvic radiotherapy
- Patients with inflammatory bowel disease
- Patients with a prior or concurrent malignancy whose natural history or treatment has the potential to interfere with the safety or efficacy assessment of ultra-hypofractionated radiotherapy
- History of bladder neck or urethral disease

### Evaluation of randomization and blinding

**Fig. 1:**
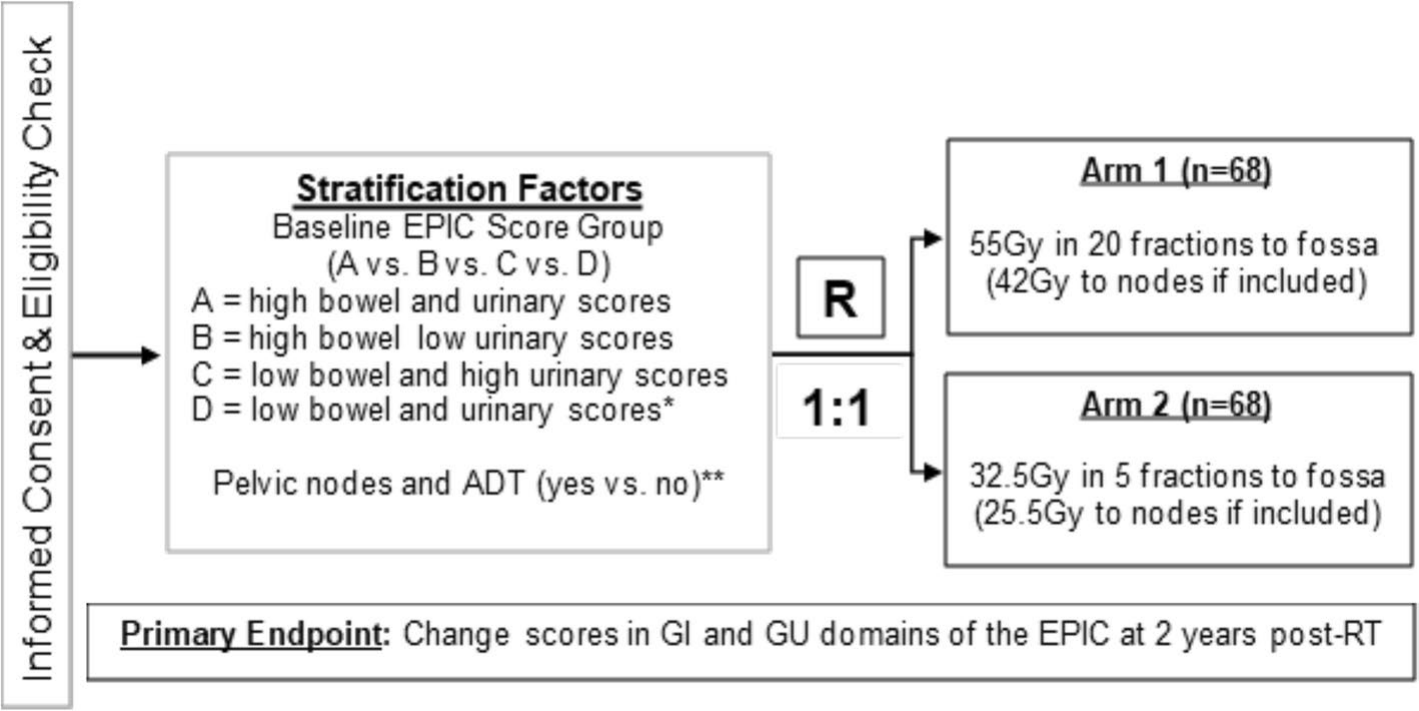
Trial Schema. Expanded Prostate Cancer Index Composite (EPIC), EuroQol-5D index (EQ-5D) and International Prostate Symptom Score (IPSS) quality of life (QoL) surveys are collected at baseline, end of salvage radiotherapy, and at 3 month, 6 month, 12 month and 60 month follow up. GI, gastrointestinal; GU, genitourinary; RT, salvage radiotherapy. *EPIC score groups defined as: high bowel score > 96, low bowel score ≤ 96, high urinary score > 84, low urinary score ≤ 84. **Patients with PSA > 0.4 ng/mL, high-risk Decipher genomic classifier scores, or pathologically node positive disease will receive pelvic nodal radiotherapy and androgen deprivation therapy (ADT) as per the clinician’s discretion

This study will employ a randomized phase II non-inferiority design to compare 5 fractions of ultra-fractionated MRgRT versus 20 fractions of moderately hypofractionated MRgRT in the salvage setting for prostate cancer patients after prostatectomy. Patients will be stratified according to baseline EPIC bowel and urinary domain scores (*i.e.,* high bowel score and high urinary score vs. high bowel score and low urinary score vs low bowel score and high urinary score vs. low bowel score and low urinary score) as well as pelvic radiotherapy and androgen therapy (*i.e.,* yes vs. no), for these factors are expected to be associated with the primary outcome. Within each strata, patients will be randomized 1:1 to receive 5 fractions or 20 fractions using permuted block randomization. The trial schema is displayed in Fig. 1.

### Interventions

#### Radiation treatment planning

After consent and eligibility verification, patients will undergo CT/MRI simulation for radiotherapy planning. Patients will receive delivery of either 32.5 Gy in 5 fractions over 2 weeks or 55 Gy in 20 fractions over 4 weeks to the prostate fossa ± pelvic lymph nodes. Indications for nodal coverage include PSA > 0.4 ng/ml, high Decipher score, or pathologically positive lymph nodes as per the clinician’s discretion [25–27]. For patients requiring nodal coverage, those on the 5 fraction arm will receive 25.5 Gy to the pelvic lymph nodes and those on the 20 fraction arm will receive of 42 Gy to the pelvic lymph nodes. Patients randomized to the 5 fraction arm cannot be treated on consecutive days. Patients undergo salvage radiotherapy no earlier than 6 months post-prostatectomy. The radiotherapy prescription doses for the 20 fraction and 5 fraction schedules are provided in Table 1.

**Table 1:** Radiotherapy Prescription Doses to Prostatic Fossa and Pelvic Lymph Nodes for 20 fraction and 5 fraction schedules

Organ	Dose (Gy)	BED (Gy)	${\mathbf{E}\mathbf{Q}\mathbf{D}}_{2}$	Dose (Gy)	BED (Gy)	${\mathbf{E}\mathbf{Q}\mathbf{D}}_{2}$	Dose (Gy)	BED (Gy)	${\mathbf{E}\mathbf{Q}\mathbf{D}}_{2}$
**PROSTATIC FOSSA**									
**Comparison of 1.8 Gy × 37 (66.6 Gy) vs. 2.75 Gy × 20 (55 Gy) vs. 6.5 Gy × 5 (32.5 Gy)**									
Prostate (α/β = 2.7)	66.6 (1.8 Gy × 37)	111	64	55 (2.75 Gy × 20)	111	64	32.5 (6.5 Gy × 5)	111	64
Bladder (α/β = 3)	66.6 (1.8 Gy × 37)	107	64	55 (2.75 Gy × 20)	105	63	32.5 (6.5 Gy × 5)	103	62
Rectum (α/β = 5)	66.6 (1.8 Gy × 37)	91	65	55 (2.75 Gy × 20)	85	61	32.5 (6.5 Gy × 5)	75	53
**PELVIC LYMPH NODES**									
**Comparison of 1.8 Gy × 25 (45 Gy) vs. 5.1 Gy × 20 (42 Gy) vs. 5.1 Gy × 5 (25.5 Gy)**									
Prostate (α/β = 2.7)	45 (1.8 Gy × 25)	75	43	42 (2.1 Gy × 20)	75	43	25.5 (5.1 Gy × 20)	74	42

##### Contour

All contouring will be done as per RTOG consensus recommendations for the prostate bed and normal pelvic structures [28]. The planning target volume (PTV) expansion for the CTV will be 2-3 mm depending on physician discretion. The bladder should not be included in the CTV (bladder overlap may be removed from the CTV via Boolean function).

##### Treatment dose planning parameters for 32.5 Gy in 5 fractions

The planning target volume (PTV) will receive the prescribed dose of 32.5 Gy in 5 fractions. The volume of PTV receiving the prescription dose (VPrescription Dose) of 32.5 Gy should be ≥ 95% and not exceed 110% (hotspot) (see treatment dose planning parameters listed in Additional File 1)*.*

##### Treatment dose planning parameters for 55 Gy in 20 fractions

The planning target volume (PTV) will receive the prescribed dose of 55 Gy in 20 fractions. The volume of PTV receiving the prescription dose (VPrescription Dose) of 55 Gy should be ≥ 95% and not exceed 115% (hotspot) (see treatment dose planning parameters listed in Additional File 1)*.*

##### Adaptive planning for 5 fraction arm

Adaptive planning will be permitted for the 5 fraction arm.

1. Prior to treatment, each patient will undergo an MRI scan.
2. 2D shifts will be performed to align relevant anatomy (bladder wall, rectum, prostatic fossa).
3. Simulation contours will be rigidly copied to the patient's MRI scan. If delineation changes, the scan will be recontoured.
4. Predicted dose algorithm will determine if treatment dose parameters meet planning dose parameters.
5. Patients will undergo adaptive planning if the treatment dose parameters do not meet the planning parameters and per the protocol planning parameters as outlined (see Additional File 1).

##### General concomitant medication and supportive care guidelines

Androgen deprivation therapy (ADT) and pelvic nodal irradiation will be administered for patients with PSA > 0.4 ng/ml, high Decipher score (> 0.6), or pathologically positive lymph nodes [25–27]. ADT should consist of a luteinizing hormone-releasing hormone (LHRH) agonist or antagonist and be initiated prior to and within 6 months of starting radiotherapy and not exceed 24 months in duration.

#### Trial Procedures

The following is an outline of the procedures to be performed at each patient visit (see Table 2):

1. Screenings
  Informed consent
  Demographics/medical history
  Physical exam
  Vital signs, height, weight
  Post-prostatectomy PSA
  Pelvic MRI
  Bone or PET scan
  Prostate 22-gene test
  Urodynamic testing (optional)
  EPIC
  International Prostate Symptom Score (IPSS)
  European Quality of Life 5 Dimension (EQ-5D)
  Common Terminology Criteria for Adverse Events version 5.0 (CTCAE v5.0)
2. First Day of RT
  Whole blood, serum, plasma
  EPIC
  IPSS
  EQ-5D
  CTCAE v5.0
3. Last Day of RT
  Whole blood, serum, plasma
  EPIC
  IPSS
  EQ-5D
  CTCAE v5.0
4. Follow-up at 3 months
  Physical exam
  Post-prostatectomy PSA 
  EPIC
  IPSS
  EQ-5D
  CTCAE v5.0
5. Follow-up at Q6 months for 2 years
  Physical exam
  Vital signs, height, weight
  Post-prostatectomy PSA
  Urodynamic testing (optional)
  EPIC
  IPSS
  EQ-5D
  CTCAE v5.0
6. Follow up at Q12 months for 3 years
  Physical exam
  Vital signs, height, weight
  Post-prostatectomy PSA
  Urodynamic testing (optional)
  EPIC
  IPSS
  EQ-5D
  CTCAE v5.0

**Table 2:** Schedule of trial events

Procedure	Screening	First Day of RT	Last Day of RT	FUP at 3mo post-RT	FUP at 6/12/18mo post-RT	FUP at 36/48/60mo post-RT
Informed Consent	X					
Demographics/Medical History	X					
Physical Exam	X			X	X	X
VS, Height, Weight	X			X	X	X
Post-Prostatectomy PSA	X			X	X	X
Pelvic MRI	X					
Bone or PET Scan	X					
Prostate 22-Gene Test	X					
Whole Blood, Serum, Plasma		X	X			
Urodynamic Testing (optional)	X				X	X
EPIC	X	X	X	X	X	X
IPSS	X	X	X	X	X	X
EQ-5D	X	X	X	X	X	X
CTCAE v5.0	X	X	X	X	X	X

*RT* salvage radiotherapy, *FUP* follow up visit, *mo* months, *CTCAE* Common Terminology Criteria for Adverse Events version 5.0

#### Definition of disease assessments

- Biochemical failure: two definitions of biochemical failure will be assessed:
  - ◦ Primary: PSA ≥ 0.4 ng/mL and rising or initiation of salvage hormones
  - ◦ Alternate: PSA ≥ PSA nadir + 2 ng/mL where nadir is the lowest post-RT PSA level
- Local failure: development of a new biopsy-proven mass in the prostate bed, after enrollment in the protocol
- Regional failure: radiographic evidence (CT or MRI) of lymphadenopathy within the pelvis (lymph node size ≥ 1.5 cm in the short axis) in a patient without the diagnosis of a hematologic/lymphomatous disorder associated with adenopathy
- Distant metastases: radiographic evidence of hematogenous spread (*e.g.,* bone scan, CT, MRI)
- Progression: first occurrence of biochemical failure, local failure, regional failure, distant metastasis, initiation of salvage ADT

#### Duration of follow Up

Patients will be followed for 5 years after removal from study or until death, whichever occurs first. Patients removed from study for unacceptable adverse events will be followed until resolution or stabilization of the adverse event.

### Statistical analysis

#### Sample size and accrual

The primary objective of this study is to determine if 5 fractions of MRgRT does not increase GI or GU toxicity over 20 fractions of MRgRT. The primary endpoints are change scores in the bowel (GI) or urinary (GU) domains of the EPIC. The change scores will reflect the 2-year score minus the pre-treatment (baseline) score. It is hypothesized that the EPIC mean change score will be no worse in the 5-fraction arm than it is in the 20-fraction arm for both GI and GU toxicity. The sample size is calculated based on a non-inferiority design. The non-inferiority margins are set to be a change score of 6 points for the GI symptoms and 5 points for the GU symptoms. The standard deviation of the change scores are assumed to be 13.2 for the GI symptoms and 10.5 for the GU symptoms based on estimates in the RTOG 0415 trial [29]. This level of change in scores seems to be clinically meaningful. A sample size of 122 with 61 in each arm will provide 80% power for the GI endpoint and 83% power for the GU endpoint to detect non-inferiority using a one-sided, two-sample t-test with 0.05 level significance. Adjusting for a projected 10% EPIC/non-compliance rate, we will accrue and randomize a total of 136 patients (68 per arm). The primary endpoint analysis will occur approximately 4 years after study activation.

#### Data analysis

##### Analysis of primary endpoints

The co-primary endpoints are GI and GU toxicity as measured by the bowel and urinary EPIC domains, respectively. The change scores, calculated as baseline score subtracted from 2-year score, will be analyzed using a non-inferiority t-test based on the prespecified non-inferiority margins with a significance level of 0.05. If the data are determined to be non-normal, a Wilcoxon test may be used instead. All patients with EPIC bowel and urinary domain scores will be included in the primary endpoint analysis. The EPIC scoring manual will be followed which requires ≥ 80% of items in a domain to be completed in order to obtain a score for that domain.

##### Analysis of secondary endpoints:

- Secondary safety endpoints: Between treatment arms differences in each safety endpoints measured as change in domain specific EPIC scores at a specific time points (*i.e.,* end of RT, 3, 6, 12, 24, and 60 months) from base line will be evaluated using t-test or Wilcoxon rank sum test whichever is more appropriate. The domain specific EPIC scores measured over time will also be modeled using a linear mixed effects model with fixed effects including time points, treatment arm, Gleason score, baseline PSA, T-stage, age, race, and a random intercept. Between treatment arms differences in GI and GU EPIC scores at 2 years adjusting for baseline scores and other covariates will be assessed using analysis of covariance (ANCOVA). Results from the primary unadjusted analysis and covariates adjusted analysis are expected to lead to similar conclusions. Otherwise, further investigation concerning possible heterogeneous subgroup effects and/or the impacts of missing values will be carried out to ensure that meaningful conclusions can be made.
- Secondary efficacy endpoints: For competing-risk endpoints such as PCSM, local failure (LF), regional failure (RF), TTP, and DM, Gray’s cumulative incidence method will be used with death as a competing risk for LF, RF and DM and death not due to prostate cancer for PCSM and TTP. OS and FFBF will be estimated by the Kaplan–Meier method and compared between arms with the log-rank test. Cox regression will be used to obtain hazard ratios (HRs) for OS and TTP. Fine and Gray’s regression will be used for the endpoints with competing risks. Adjusted HRs and the respective 95% confidence interval will be computed. Baseline PSA, stratification variables (baseline EPIC score and ADT status), age, race, and other covariates (Gleason, T-stage), will be adjusted for as appropriate in this analysis.

##### Early stopping guidelines

An interim futility analysis will be conducted when one-third of patients have had their 6 months follow-ups. If the upper 95% confidence limit of the mean difference in 6-months change scores between the treatment arms is less than the pre-specified non-inferiority margins (*i.e.,* <-6 for GU and <-5 for GI), then the 5-fraction arm will be deemed inferior to the 20-fraction arm and the study will be halted.

## Discussion

With growing acceptance of hypofractionation and the increase of ultra-hypofractionation for definitive radiation treatment of intact prostate cancer, hypofractionated regimens for post-operative management are poised to become a convenient option for patients. Abbreviated radiation schedules may reduce the burden of treatment on patients and treatment centers alike while maintaining clinical efficacy and safety. A primary consideration for patients in need of post-operative radiotherapy is toxicity, manifesting as both acute and late side effects of the bladder and bowel. Such side effects impact quality of life following treatment, highlighting the importance of treatment approaches that minimize toxicity. MRgRT may facilitate higher radiation doses to be delivered in fewer sessions with increased accuracy and precision. The potential advantages of MRgRT include better visualization of the prostatic fossa, the capacity for real-time tracking, and the ability to perform online adaptive radiotherapy that accounts for organ deformation. These factors may decrease the radiation dose received by adjacent structures, thereby minimizing toxicity [23, 24]. The SHORTER trial is the first randomized trial to evaluate whether the theoretical advantages of hypofractionation combined with MRI-guidance translate into the salvage radiotherapy setting from a toxicity standpoint. The SHORTER trial will provide information not only on toxicities, but also well-documented information on time to progression, prostate cancer-specific survival and overall survival. We hope that the data from this trial will elucidate the non-inferiority of ultra-hypofractionated, MRI-guided post-operative radiotherapy.

### Supplementary Information

**Additional file 1.**

## Data Availability

The datasets generated and/or analyzed during the current study are not publicly available due to the ongoing nature of the trial and possible compromise of individual privacy but are available from the corresponding author upon reasonable request.

## Abbreviations

SBRT: Stereotactic Body Radiotherapy
MRI: Magnetic Resonance Imaging
OAR: Organs at Risk
MRgRT: MRI-guided Radiotherapy
GU: Genitourinary
GI: Gastrointestinal
BCR: Biochemical recurrence
PSA: Prostate Specific Antigen
MR-LINAC: Magnetic Resonance Linear Accelerator
CT: Computed Tomography
EPIC: Expanded Prostate Cancer Index Composite
TTP: Time to progression
DM: Distant metastasis
PCSM: Prostate cancer-specific morbidity
FFBP: Freedom from biochemical progression
OS: Overall survival
PET: Positron Emission Tomography
PTV: Planning Target Volume
LHRH: Luteinizing hormone-releasing hormone
IPSS: International Prostate Symptom Score
EQ-5D: European Quality of Life 5 Dimension
CTCAE v5.0: Common Terminology Criteria for Adverse Events version 5.0
ADT: Androgen Deprivation Therapy
LF: Local failure
RF: Regional failure
HR: Hazard ratio
