# Supplementary material for: Randomized phase II trial of MRI-guided salvage radiotherapy for prostate cancer in 4 weeks versus 2 weeks (SHORTER)
Source: BMC Cancer. 2023 Aug 22;23:781. doi: 10.1186/s12885-023-11278-3 (PMC10463903; doi:10.1186/s12885-023-11278-3)
Supplement: Supplementary file 1 — Additional file 1. [file 12885_2023_11278_MOESM1_ESM.pdf]

Supplemental Table 1. Radiotherapy Treatment Constraints and Planning Parameters

	**5 FRACTION ARM**	
**Structure**	**Dosimetric Parameter**	**Per Protocol**
Rectum	V25	< 20 cc
	Max Point Dose	< 38 Gy
Bladder Wall	V18.3	<15 cc
	V25	< 18 cc
	Max Point Dose	< 38 Gy
Femoral Heads	V30	< 10 cc
	**20 FRACTION ARM**	
**Structure**	**Dosimetric Parameter**	**Per Protocol**
Rectum	V24	< 80 cc
	V32	< 70 cc
	V40	< 60 cc
	V48	< 50 cc
	V55	< 40 cc
Bladder Wall	V40	< 80 cc
	V48	< 50 cc
Femoral Heads	D44 Gy[%]	< 10
